# Flax, Basalt, E-Glass FRP and Their Hybrid FRP Strengthened Wood Beams: An Experimental Study

**DOI:** 10.3390/polym11081255

**Published:** 2019-07-29

**Authors:** Bo Wang, Erik Valentine Bachtiar, Libo Yan, Bohumil Kasal, Vincenzo Fiore

**Affiliations:** 1 Department of Organic and Wood-Based Construction Materials, Technische Universität Braunschweig, Hopfengarten 20, 38102 Braunschweig, Germany; 2 Center for Light and Environmentally-friendly Structures, Fraunhofer Wilhelm-Klauditz-Institut, Bienroder Weg 54E, 38108 Braunschweig, Germany; 3 Department of Engineering, University of Palermo, Viale delle Scienze, Edificio 6, 90128 Palermo, Italy

**Keywords:** flax FRP, basalt FRP, glass FRP, wood beam, bending, hybrid FRP

## Abstract

In this study, the structural behavior of small-scale wood beams externally strengthened with various fiber strengthened polymer (FRP) composites (i.e., flax FRP (FFRP), basalt FRP (BFRP), E-glass FRP (“E” stands for electrical resistance, GFRP) and their hybrid FRP composites (HFRP) with different fiber configurations) were investigated. FRP strengthened wood specimens were tested under bending and the effects of different fiber materials, thicknesses and the layer arrangements of the FRP on the flexural behavior of strengthened wood beams were discussed. The beams strengthened with flax FRP showed a higher flexural loading capacity in comparison to the beams with basalt FRP. Flax FRP provided a comparable enhancement in the maximum load with beams strengthened with glass FRP at the same number of FRP layers. In addition, all the hybrid FRPs (i.e., a combination of flax, basalt and E-glass FRP) in this study exhibited no significant enhancement in load carrying capacity but larger maximum deflection than the single type of FRP composite. It was also found that the failure modes of FRP strengthened beams changed from tensile failure to FRP debonding as their maximum bending load increased.

## 1. Introduction

With an increasing concern on the energy conservation and environment protection, wood as a natural and sustainable construction material has returned to the spotlight after a long time flagging [1]. Compared with other conventional construction and building materials, wood has several shortcomings, e.g., relatively low tensile stiffness and strength compared to steel and low compression stiffness and strength compared to concrete. Wood is also susceptible to biological degradations, such as from fungi, bacteria and insects [2], which weaken its mechanical properties. To overcome the inferior mechanical properties of wood elements, fiber reinforced polymer (FRP) composite [3,4,5] can be one of the solutions. FRP has been widely utilized in the past two decades for rehabilitation and reinforcing of existing structures. FRP materials such as glass or carbon FRP have high strength-to-weight ratio, corrosion-resistance and provide design flexibility [6,7,8]. 

The commonly utilized FRP composites as reinforcement for wood beams are carbon FRP (CFRP), E-glass FRP (GFRP) and aramid FRP (AFRP) [3,4,5,9,10,11,12]. However, the production processes of these fibers are energy-intensive and the initial costs are still high. Recently, mineral-based natural FRP, such as basalt FRP (BFRP), has been introduced. BFRP has low material cost, high fire resistance, good thermal, electrical and sound insulating properties [13,14,15]. Furthermore, basalt fiber also has high tensile properties (e.g., tensile strength of 1850–4800 MPa) [14]. However, similar to glass fiber, the production of basalt fiber also requires a large amount of energy because of the high melting point of basalt rocks (1300 °C–1700 °C) [13].

As an alternative to glass, carbon and basalt fiber materials, the ecological and economical plant-based FRPs (e.g., flax or jute FRP) have been introduced in civil engineering. Various investigations on plant-based fibers (e.g., flax) have shown that as a single fiber, they have comparable specific mechanical properties (e.g., specific tensile strength and stiffness) compared to those of man-made E-glass fiber [6]. However, this is somewhat misleading since the length of natural fibers are limited, while carbon or glass fiber can be manufactured to have an endless length. The natural fibers are used in the forms of yarns, which will generally have lower mechanical properties compared to the ones of individual fibers.

Nevertheless, several investigations using the natural fibers in FRP as a reinforcement in civil engineering application have been carried out. Huang et al. [16] investigated flax FRP (FFRP) strengthened reinforced concrete (RC) beams. Their results revealed that the FFRP increased the ultimate load and maximum strain as well as the ductility of RC beams significantly. It also showed a better interfacial compatibility with the RC beams compared to GFRP and CFRP strengthened RC beams. Yan et al. [17] investigated the flexural properties of plain concrete beams externally strengthened with FFRP. It has been shown that the bending load capacity of plain concrete beams increased by 100%, 230% and 327% and their fracture energy were increase by 3500%, 4200% and 8160% with two-, four- and six-layer FFRP reinforcement [17]. In addition, FFRP has been used as external confining materials of natural aggregate concrete [18], recycled aggregate concrete [19] and fiber reinforced concrete [20,21].

In literature, a large number of studies have investigated FRP as an external reinforcement of wood structures, but only very few have considered plant-based FRPs. For example, Speranzini et al. [22] investigated solid wood beams externally strengthened with carbon, glass, basalt, hemp and flax FRP under a four-point bending test. No significant difference was observed on the loading capacity of the different FRP composites (i.e., the increase of the bending strength were 42.3%, 24.6%, 23.2%, 24.0% and 35.4% for carbon, glass, basalt, hemp and flax FRP, respectively) although there was a large difference in the tensile strength of these FRPs (i.e., 479, 142, 245, 36 and 25 MPa for carbon, glass, basalt, hemp and flax FRPs, respectively). According to the author, flax and hemp fibers may have better adhesion to wood compared to other FRPs. Borri et al. [23] investigated flax and basalt FRP strengthened low-grade (bending strength of 18.4 MPa) and high-grade (bending strength of 41.3 MPa) wood beams. The tensile strengths of FFRP and BFRP in the study was 240 MPa and 1880 MPa, respectively. The results showed an increase of bending strength of 38.6% and 65.8%, and maximum mid-span deflection of 58.2% and 40.2% respectively by two-layer FFRP and BFRP strengthened low-grade wood beams. Moreover, the strength increases were 29.2% and 25.9%, the increases of maximum mid-deflection were 9.1% and 14.5% respectively for two-layer FFRP and BFRP strengthened high-grade wood beams. This study concluded that both BFRP and FFRP provided the beams with higher strength and better ductile behavior. Similar results can be found in another research by Borri et al. [24] for flax and basalt FRP. André et al. [25] applied FFRP and GFRP with similar fabric density (i.e., $230\;g/m^{2}$ for flax and $250\;g/m^{2}$ for glass) perpendicular to grain on wood beams. It is reported that the maximum bending load of the entire specimen strengthened with GFRP (45.1 kN) was 23% higher than that one strengthened with FFRP (36.0 kN).

Realizing the advantages and disadvantages of using different types of fibers in FRP, hybrid FRP (HFRP) was proposed in the literature. Hybrid FRP, which consists of two or more combinations of strengthened fibers or fabrics, was designed to inherit the advantages and minimize the disadvantages of the combined fibers. Kim et al. [26] investigated HFRP made of carbon and glass fabrics to retrofit RC beams. The results showed that the HFRP contributed to higher ultimate bending strength and ductility of the RC beams compared to the single type of CFRP or GFRP. The maximum load in bending of RC strengthened with GFRP–CFRP (G GFRP attached at the tension surface of the RC beam) specimens was 6.6% and 3.9% higher than the one strengthened with two-layer CFRP (CC) and two-layer GFRP (GG), respectively. Moreover, the maximum mid-span deflection was also 27.4% and 18.5% higher than that of CC and GG specimens. 

Compared with man-made fiber/fabric materials in conventional FRP composites (e.g., E-glass and carbon), plant-based fiber/fabric has a lower price and positive ecological impact [27], but it has lower mechanical properties as it has been mentioned before. In order to balance the performance and the cost for proper material design, several studies have investigated the hybridization of a plant-based fabric with a man-made one in FRP composite [28,29]. Gupta et al. [29] have summarized the mechanical properties of this hybrid material reinforcing thermoset polymers. It was concluded that the tensile, flexural and impact strengths of hybrid FRP were higher than those of the single type natural fabric FRP. However, the application of the hybrid FRP with natural fabric for reinforcing wood beams have been scarcely investigated before. Throughout the literature, only very few studies have investigated HFRP strengthened wood beams. Yang et al. [30] strengthened wood beams with hybrid carbon and glass FRP. Compared to the wood beams strengthened by GFRP or CFRP alone, the HFRP provided a larger energy dissipation for wood beams.

In this study, the flexural behavior of flax FRP strengthened wood beams were investigated. The results were compared with man-made E-glass and mineral-based basalt FRPs. Additionally, hybrid flax/glass/basalt FRPs were also investigated and compared with single type of FRPs (i.e., FFRP, BFRP and GFRP). Various different FRP materials (i.e., FFRP, GFRP and BFRP), FRP thickness (i.e., one-, two- and three-layer) and the arrangement of FRP in the HFRP were considered as experimental variables. As complementary initial investigations, tensile and bending test of flat coupon single type fiber FRPs were also carried out. Furthermore, since the interfacial bonding of fiber/epoxy and FRP/wood are also critical points for the flexural behavior of beams, the microstructures of these interfaces from the fractured specimens were examined under light and scanning electron microscopes.

## 2. Materials and Methods

### 2.1. Materials

Flax, basalt and E-glass were selected to represent the plant-based, mineral-based and conventional man-made fiber/fabric material for FRP composites, respectively. Among plant-based fibers, flax has comparable specific tensile properties with a lower unit price compared to those of E-glass fiber [6]. In addition, flax has a short growing cycle (harvested within 100 days after sowing the seeds). It also has a large annual production, which is required due to its broad applications, e.g., for household textiles, sails or tents, etc. [6]. For mineral-based fibers, basalt is generally used as a replacement of dangerous asbestos fibers and probably the only mineral-based fiber type that is available on the market [27]. Furthermore, basalt fiber also has tensile properties close to those of carbon fibers (e.g., for tensile strength, basalt fiber: 1850–4800 MPa and carbon fiber: 3000–5000 MPa) [14]. E-glass is one of the most widely used fibers as it is cheaper than carbon or aramid fibers and it has relatively high tensile strength (1800–3500 MPa).

**Figure 1:**
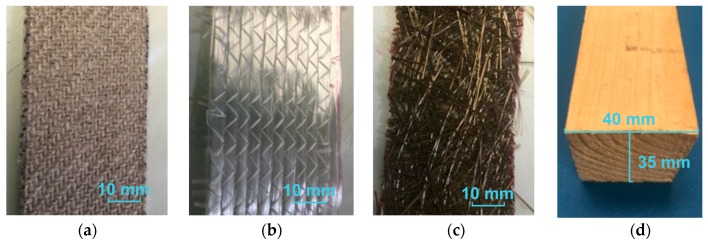
Photos of testing materials: (**a**) flax fabric, (**b**) glass fabric, (**c**) basalt mat and (**d**) wood beam.

In this study, bidirectional woven flax fabric (FlaxPly BL 550 from Lineo, Valliquerville, France, seven single-strand yarn threads per cm in the fabric weft and warp directions) (Figure 1a), unidirectional E-glass fabric (S15EU910, Saertex GmbH & Co. KG, Saerbech, Germany) (Figure 1b) and randomly distributed basalt mat (HG Europe, Milano, Italy) (Figure 1c) were investigated as FRP fabric materials. Based on the supplier data sheets, the areal density of flax, E-glass and basalt fabrics are 550 g m^−2^, 600 g m^−2^ and 220 g m^−2^, respectively. The nominal fiber thicknesses for one layer of flax, basalt and glass fabrics were 1.2 mm, 0.7 mm and 0.9 mm, respectively. However, it has to be mentioned that these nominal fiber thicknesses were only rough approximations as they are highly dependent on the pressure applied during measurement and the weaving structure of the fabrics. The FRPs were manufactured with a two-component epoxy polymer PRIME^TM^ 20LV epoxy resin and Prime 20 Slow hardener by Gurit Company, Zullwil, Switzerland. The tensile strength, tensile modulus and strain at failure of the cured epoxy were 73 MPa, 3.5 GPa and 3.5%, respectively. Although, some other adhesives (such as phenolic [31,32] or melamine [33] based adhesives), which are commonly used as adhesives for wood or other cellulosic materials, can be used as a matrix. Epoxy resin was selected in this study since it has been proven to have higher mechanical properties and chemical resistance than the other adhesives [6,34]. Epoxy is also the most commonly used polymer in FRP composites [7,8,20]. The structural wood beams, which were strengthened by the FRPs, were manufactured from Douglas Fir (*Pseudotsuga menziesii* Mirb.) with a dimension of 600 mm (length) × 40 mm (width) × 35 mm (height). The length direction of the beam was along the fiber direction of the wood (Figure 1d). The average density of the wood beams was 577 ± 33 kg·m^−3^.

### 2.2. Manufacture of FRP and FRP–Wood Specimens

The FRP manufacture process in this study was conducted through hand wet lay-up process and two kinds of specimens were produced: (1) FRP laminates for tensile and bending test and (2) FRP strengthened wood beams for bending tests. Initially, the epoxy resin and hardener were mixed with a ratio of 1:0.26 by weight for five minutes. The first layer of the fabric was placed on a flat and water-proofed plastic foil surface. It was then saturated with the epoxy mixture by using a brush. To avoid excess epoxy resin on the fabric, the saturation process was conducted slowly and directly stopped as soon as the fabric reached the saturation point. After that, the next layer was laid on the top of the first one and slowly saturated again with the epoxy. This process was repeated until the targeted number of layers was reached. Similar steps were used for the hybrid FRP. The fabrics were laid one by one in the intended order. All the epoxy-impregnated FRP composites were then cured at a room temperature (20 ± 3 °C) for seven days before they were cut to laminates for the flat-coupon tensile and flexural tests. No external pressure was applied on the FRP composites during the curing process. For tensile and bending tests, the FRP was cut into the appropriate size after curing. For the production of FRP strengthened wood specimens, the fabrics were cut firstly into strips with the size of 600 mm × 40 mm and the surface of the beams were coated by epoxy. Then, the strips were applied directly on the wood beams. While the basalt mat was arbitrarily applied on the wood beam due to its random orientation, the main fiber direction of the glass fabric and the warp direction of the flax fabric were always applied along the grain of the wood.

### 2.3. Test Matrix

A total of 39 small-scale wood beam specimens (three wood beams and 36 FRP strengthened wood beams) were tested under a three-point bending test according to DIN 52186 [35]. Table 1 shows the test matrix of the specimens used in this study. In the specimen name for each specimen type, W indicates wood, while F, B and G denote flax, basalt and glass as the type of the fabric for the FRP composites, respectively. The number of the FRP layers are denoted by 1L, 2L and 3L, i.e., one-, two- and three-layer. For hybrid FRP composite strengthened wood, the combination of F, B and G denotes the sequence of the arrangement of the FRP composite, i.e., 3L-GBF indicates the arrangement of the FRP, which is the outer layer (glass), middle layer (basalt), and the inner layer (flax) attached to the wood beams.

The mechanical properties of the different FRP composites were determined before the bending test of FRP-wood beams. Flat coupon tensile and bending tests were carried out for the FRP laminates according to ASTM D 3039 [36] and ASTM D 790 [37], respectively. For both tests, FRP composites with three different fabric materials (i.e., flax, glass and basalt) and three different layers (i.e., one-, two- and three-layer) were tested. For each specimen type, 10 specimens were prepared with the size of 250 mm in length × 25 mm in width and 150 mm in length × 25 mm in width for tensile and bending tests, respectively. The final thicknesses of the FRP laminates were determined by averaging the thickness of the laminates at three different locations. These thicknesses are presented as results in Table 2.

### 2.4. Test Instrumentation

**Figure 2:**
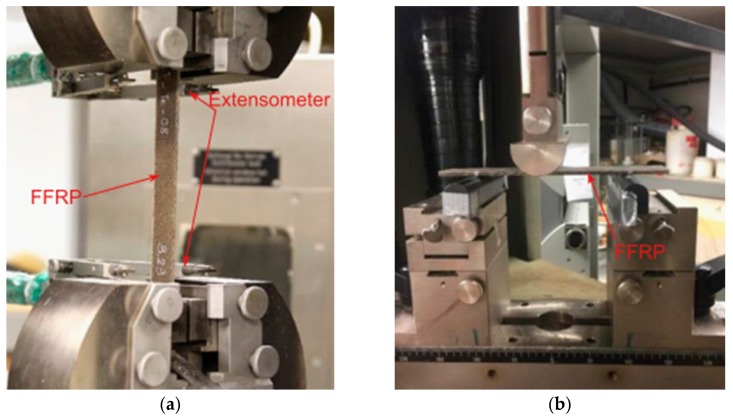
(**a**) Flat coupon tensile test and (**b**) bending test for fiber reinforced polymer (FRP) laminates.

**Figure 3:**
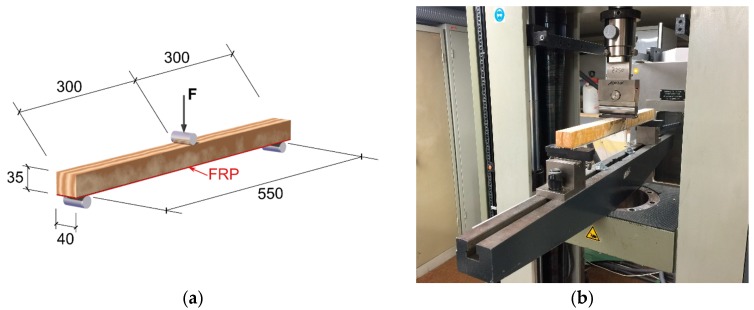
Test setup of bending test for FRP–wood beams (unit: mm).

Zwick 1474 Test Machine (from ZwickRoell GmbH & Co. KG, Ulm, Germany) with a load cell capacity of 100 kN was used for flat coupon tensile test (Figure 2a), bending test (Figure 2b) for FRP laminates and three-point bending test for FRP strengthened wood beams (Figure 3). The testing machine was equipped with a standard extensometer (with an initial distance of 140 mm) to record the displacement of the sample during the test. The tensile tests were carried out with a displacement-controlled rate of 2.5 mm/min. The bending tests on FRP laminates were performed with a span distance of 100 mm and based on the standard, the testing rate was calculated as:

(1) $$R=ZL^{2}/6d$$

where,
Rrate of crosshead motion, mm/minZrate of straining of the outer fibric, 0.01%/minLsupport span, mmdthickness of the specimen, mm.

All tests for FRP laminates were conducted until failure or the maximum strain of 5% was reached.

The span of FRP strengthened wood beams tested under bending loading was 550 mm. The load was applied at the middle of beams with a loading rate of 12 mm/min until failure. The apparent flexural elastic modulus of the FRP–wood beams was calculated through the following equation, which is adapted from DIN 52186 [35]:

(2) $$E\;=\;\frac{L^{3}}{4bd^{3}}\cdot \frac{∆F}{∆D}$$

where
Eflexural elastic modulus, GPaLsupport span, mmbwidth of the tested beam, mmddepth of the tested beam, mm∆Fdifference of force between 20% to 40% of the maximum bending loading, kN∆Ddifference of mid-span displacement at the corresponding bending loading, mm

After the mechanical tests, the fracture areas of the FRP–wood beams were observed with a light microscope (ZEISS 47 50 57 from Carl Zeiss Jena GmbH, Jena, Germany) and a scanning electron microscope (SEM, JSM-6700F, JEOL LTD, Tokyo, Japan). The specimens for the SEM were vacuum-coated with gold by evaporation process in BAL-TEC SCD 050 sputter coater.

### 2.5. Data Analysis Method

During the analysis and the interpretation of the data, the results were only compared based on the average value. The readers must be cautioned that these comparisons were only preliminary in character due to the comparing of the average values. No statistical analysis of the data was possible due to the limited number of specimens. Matching of specimens (for a pairwise comparison) is impossible for wood samples due to the variability within the material itself as well as variability between the specimens. 

## 3. Results and Discussion

### 3.1. Tensile and Bending Tests for FRP Laminates

The results of the tested FRP laminates under tensile and bending loadings are presented in Table 2. For each specimen type, eight to ten specimens were successfully tested, except for 3L_F_Te and 2L_G_Te, where six and five specimens were successfully tested, respectively. The averaged value and the standard deviation of these successfully tested specimens are presented in the table. Furthermore, Figures S1 and S2 show the tensile and flexural stress–strain curves of the specimens during the tests, respectively. In these table and figures, indices Te and Be refer to tensile and bending tests, respectively.

Under bending loading, the maximum strengths of BFRP (79.6–156.8 MPa) were in general higher than FFRP (60.3–94.6 MPa) at any number of investigated fabric layers. Under tensile loading, however, FFRP (41.7–76.8MPa) had a comparatively similar strength than those of BFRP (49.6–61.1 MPa). Based on previous studies, the tensile strength of BFRP can be reached at around 1000 MPa (e.g., 707 MPa by Reyes-Araiza et al. [38] and 1282 MPa by Quagliarini et al. [39]). The low strength of BFRP obtained in this study was suspected due to the thin nominal fabric thickness, which led to a low areal density, and the random distribution of the basalt fibers in the mat. When compared with GFRP, FFRP presented significantly lower tensile and bending properties, and lower strain at peak load. This was expected since flax yarn consists of multiple bundles of short fibers, while glass yarn may have continuous fibers. Flax fibers may also contain natural defects [6], which cannot be avoided. Similar results were reported by Zhang et al. [40]. Their results showed that 10-layer FFRP had tensile strength of about 220 MPa and tensile failure strain of 0.85%, which was much lower than 10L-GFRP with tensile strength of about 700 MPa and tensile failure strain of 1.41%.

The number of fabric layers also influenced the mechanical properties of the overall FFRP. A relatively similar tensile strength was observed for one-layer and two-layer FFRP (41.7 and 48.2 MPa, respectively). However, the three-layer FFRP provided distinctly higher tensile strength (76.8 MPa). Under bending, on the other hand, 1L-FFRP (60.3 MPa) had a lower strength compared to the 2L- and 3L-FFRP (94.6 and 90.3 MPa, respectively). The strains at the peak load of the FFRP specimens also followed the same pattern. Under tensile loading, 3L-FFRP was observed to have a higher maximum strain (1.69%) compared to 1L- and 2L-FFRPs (1.29% and 1.30%, respectively). In contrast, the 1L-FFRP specimens had the highest strain at failure under bending load (2.26% compared to 3.37% and 3.23% for 2L- and 3L-FFRP, respectively). Besides the number of layers and the type of loading (tension or bending loadings), the inconsistency of the produced fiber volume fraction of the FRP using hand lay-up method may have contributed to the current finding. Moreover, under bending loading the thickness of the specimen strongly influenced the results. When it was bent in a same span length, a thicker specimen produced more internal shear, thus, it was stiffer and failed faster compared to a thinner specimen.

### 3.2. Bending Tests for FRP Strengthened Wood Beams

#### 3.2.1. Effect of FRP Thicknesses on the Bending Behavior of FRP Strengthened Wood Beams

Figure S3 shows the representative load–displacement curves of wood beams strengthened with a different number of layers of B-, F- and GFRP. The results together with the calculated improvement of the properties due to the FRP reinforcements (unstrengthened wood beams as the reference) are also presented in Table 3.

The load capacity improvement increased with an increasing number of FRP layers for all wood beams strengthened with a single type of FRP. FFRP strengthened wood beams had maximum bending load capacities of 4.5, 5.5 and 6.2 kN for one, two and three layers, respectively. These corresponded to 60.7%, 96.4% and 121.4% load capacity improvement compared to unstrengthened wood beams, which had an average maximum load capacity of 2.8 kN. However, the improvement of the load bearing capacity was not linearly proportional to the increasing number of FRP layers. Similar to the FFRP, the load capacity improvements of one, two and three layers of GFRP were 71.4%, 117.9% and 132.1%. As the number of FRP layers increased, the gradient of the capacity improvement declined. However, surprisingly it was observed that the gradient of the capacity improvement of BFRP increased, i.e., 14.3%, 50.0% and 107.1% for one, two and three layers of BFRP. The reason could be due to the change of the failure mode, which is often governed by the weakest components in the FRP–wood composite beams. Under bending, the load is transferred to the compression and tension loadings. The compression loading was on the top part of the specimen, which was carried by the wood, while tensile loading was on the bottom part carried by the FRP. The tensile rupture of FRP may have initiated the overall failure of the composite if the FRP laminates were too thin (e.g., the 1L-FRP) or did not have enough strength (e.g., flax and basalt). With the increasing number of layers in the FRP, the tensile capacity of the FRP increased, which may have led to a shifting of the failure mode. The FRP–wood composite may then have failed due to the yielding failure of the wood in the compression zone or the delamination of the FRP–wood interface due to the induced internal shear loading. Further discussions of the different failure modes are given in Section 3.3.

Table 3 also presents the elastic modulus of the investigated FRP–wood composites. The elastic modulus may have increase up to 66% as the woods were strengthened with the FRP. The influence of FFRP and BFRP thickness on the elastic modulus of the overall beam was less pronounced compared to the one from GFRP. By using one-, two- and three-layer GFRP under the wood beams, the elastic modulus was increased from 9.0 GPa to 9.2, 13.3, and 15.1 GPa, respectively. Among all the specimens, wood beams strengthened with three layers of GFRP had the highest elastic modulus. Compared with BFRP (10.0–11.0 GPa), FFRP strengthened wood beams had a higher elastic modulus (12.6–12.9 GPa). However, these results do not fully follow the results of the tensile tests and bending tests of FRP laminates showed in Section 3.1. FFRP laminates had the lowest tensile and bending modulus (i.e., 4.8–5.6 GPa in tensile and 3.7–5.1 GPa in bending) compared to BFRP (6.0–6.2 GPa in tensile and 5.8–6.3 GPa in bending) and GFRP (19.3–23.3 GPa in tensile and 8.0–18.1 GPa in bending). The reason of these findings was suspected to be due to the different thickness of FRP beams and the compatibility between the fabric and wood.

Based on the cross-section inertia of the beams and also presented in Equation (2), the height of the beam to the power of three highly influences the elastic modulus. The actual thickness of each specimen was considered in the calculation. However, the different thickness of the FRP led to the different height of the FRP–wood specimens. Thus, the cross-sectional FRP–wood ratios were varied between specimens. This may have led to a different stress distribution during bending loading. Higher thickness of the FRP–wood may also have resulted in stiffer beams due to the more pronounced influence from the internal shear loading of the specimen under bending loading.

In addition to that, as a cellulosic natural material, flax has the same chemical components as wood (i.e., cellulose, hemicellulose and lignin). Therefore, similar bonding behavior is expected between flax/epoxy and epoxy/wood. On the other hand, the bonding behavior of glass/epoxy and basalt/epoxy are different. The similar bonding behavior was suspected to give a positive impact of the overall mechanical properties of the FRP–wood beams. This was also supported by the results from HFRP, the highest stiffness was reached when flax connected directly to the wood (14.6 GPa for W_3L-BGF). This reason, however, is only a theory based on the results obtained in this study. Further investigations have to be conducted to support this theory.

#### 3.2.2. Effect of FRP Materials on the Bending Behavior of FRP Strengthened Wood Beams

**Figure 4:**
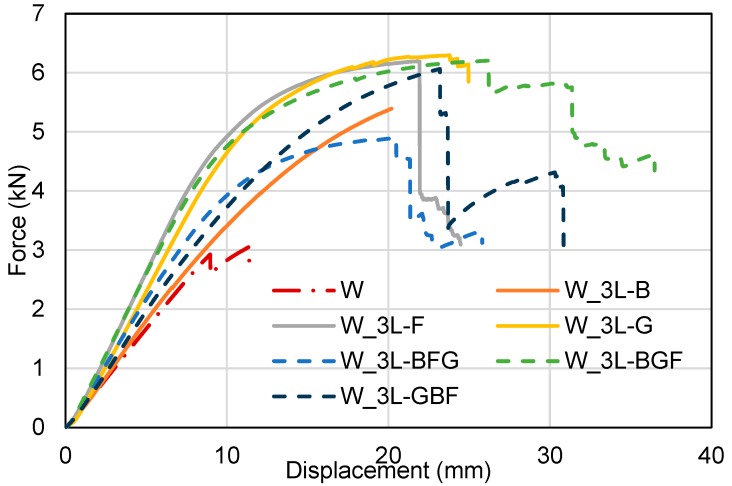
Load mid-span displacement curves of three-layer FRP strengthened wood beams.

Figure 4 shows the representative bending load–displacement curves of the unstrengthened wood beam and all types of three-layer FRP strengthened wood beams. Their maximum load, maximum deflection and flexural elastic modulus are presented in Table 3. All the three-layer FRP reinforcements increased the maximum load of wood beams remarkably. The average load capacity of W_3L-F, W_3L-B and W_3L-G were 6.2, 5.8 and 6.5 kN with increments of 121.4%, 107.1% and 132.1%, respectively in comparison to the average load capacity of unstrengthened wood beams. The hybrid FRPs showed similar enhancement in load capacity. The maximum bending load of W_3L-BFG, W_3L-BGF and W_3L-GBF were 5.6, 5.8 and 5.9 kN, respectively. Among these tested FRPs, the best performance based on the maximum mid-span deflection was observed from HFRP strengthened wood beams. The W_3L-BGF had the highest maximum strain increment by 142.5%, followed by W_3L-GBF (134.6%) and W_3L-BFG (121.3%), which were higher than that of FFRP (66.9%), BFRP (63.0%) and GFRP (111.0%).

When comparing the FFRP to BFRP and GFRP, it was found that FFRP laminates had higher ultimate strain than BFRP under tensile loading. Therefore, FFRP provided a larger enhancement in deflection than BFRP for FRP strengthened wood beams. FFRP had only a slightly lower tensile strength to BFRP (41.7 and 49.6 MPa for one-layer FFRP and BFRP, respectively), which was already enough to carry the tensile loads on the tensile area at the bottom of the wood beams. Moreover, FFRP laminates were also thicker than BFRP. As a result, FFRP provided larger enhancement than BFRP in FRP strengthened wood beams. It should be, however, kept in mind that the basalt fabric mat used in this study had a low areal density with short fibers that were orientated randomly. Furthermore, the bending results of FRP strengthened wood beams also showed that FFRP provided similar maximum strength and maximum deflection enhancement with GFRP for wood beams (especially with higher number of FRP layers), although FFRP laminate had much lower tensile strength than GFRP laminates (i.e., 76.8 MPa for three-layer FFRP vs. 449.1 MPa for three-layer GFRP). This was primarily because, at a high number of FRP layers, the failure of the interface between wood and epoxy would have been more decisive on initiating the whole failure of the FRP–wood beams. Thus, having a stronger FRP material such as glass, may not necessarily increase the overall performance of FRP–wood composite. The interface debonding will always initiate failure of the whole composite systems and the maximum capacity of GFRP cannot be fully utilized.

### 3.3. Failure Modes and Microstructure of FRP–Wood Beam System

#### 3.3.1. Failure Modes

**Figure 5:**
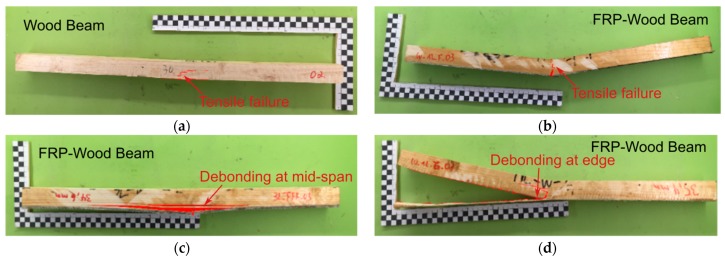
Typical failure modes of FRP–wood beams under three-point bending with different FRP laminates: (**a**) tensile failure for reference wood beam, (**b**) tensile failure (W_1L-F), (**c**) debonding at mid-span (W_3L-F) and (**d**) debonding at edge for FRP strengthened wood beam (W_1L-G).

The typical failure modes of the reference wood beams and FRP strengthened beams are shown in Figure 5. The reference beams showed a typical tension failure (Figure 5a). The crack was initiated at the mid-span of the tensile zone and then propagated until the complete failure of the beam. For FRP strengthened wood beams, two kinds of failure were observed, i.e., tensile failure and debonding of FRP. The tensile failure in FRP–wood beams (Figure 5b) was initiated at the middle of FRP strips followed by the failure of the tensile zone of wood beams. The debonding of FRP took place at the interface between wood beams and FRP and occurred in either mid-span of the beam (Figure 5c) or at the edge (Figure 5d).

Table 4 presents the general failure modes for all specimens tested in this study. It can be observed that most FRP strengthened wood beams with low maximum bending loading (e.g., less than 5.8 kN) showed a primarily tensile failure mode. At a higher bending loading, interface debonding was observed. The reason has already been discussed previously that weakest parts (between wood, FRP and the FRP–wood interface) of the composite will decide the failure mode. Thus, by changing of the number of layers the failure mode may be also changed. Exception can be found in W_1L-G with maximum bending load of 4.8 kN, where debonding failure was observed and W_3L-B with maximum bending load of 5.8 kN, where tensile failure was observed. W_3L-GBF had also a slightly higher maximum bending load of 5.9 kN, but tensile failure was observed This may have been due to the relatively low manufacturing quality and repeatability through the hand wet lay-up process. Further investigations on the relation between the failure mode and the tensile strength of FRP strengthened beams should be carried out in the future

#### 3.3.2. Microstructure

**Figure 6:**
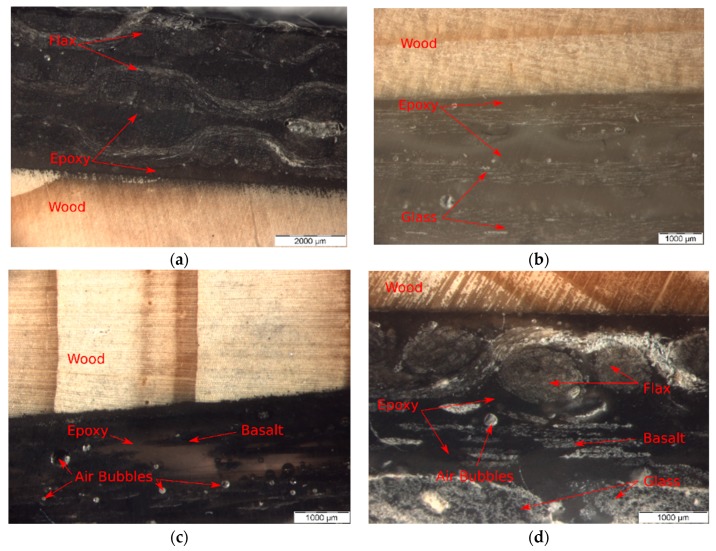
Light microstructure of (**a**) flax (F)FRP–wood, (**b**) E-glass (G)FRP–wood, (**c**) basalt (B)FRP–wood and (**d**) hybrid FRP–wood.

Figure 6 shows the light microstructures of FFRP, GFRP, and BFRP as well as a hybrid FRP strengthened wood beams (W_3L-GBF). The interface between epoxy/wood, flax yarn, glass and basalt fiber structure can be clearly observed under the light microscope. As can be seen, no gaps were found in the interfaces between epoxy and wood. Such interface eased the transfer of the bending load from wood to the FRP fabric. However, several air bubbles were observed in the FRP. These air bubbles might be regarded as defects which may result in stress concentration at the FRP/wood interface. This should be further identified in a future study. The presence of the air bubbles may explain why W_1L-G had a lower bending load of 5.4 kN with debonding failure compared to W_3L-B and W_3L-GBF.

**Figure 7:**
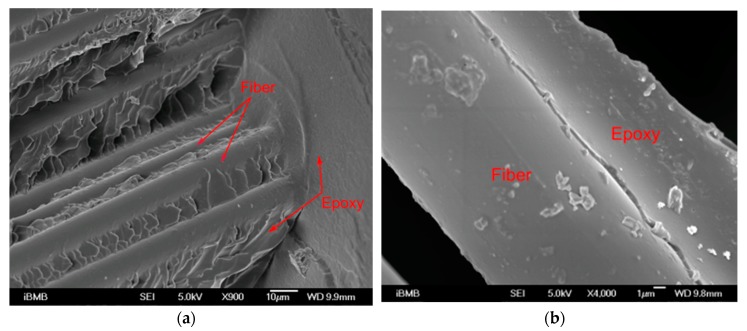
The fracture surface from SEM of (**a**) the interface between epoxy and basalt fiber, (**b**) a close-up of the interface.

The scanning electron microscope analysis (SEM) was used for the observation of the interface of fabric/epoxy (or fiber/epoxy). Figure 7 shows the example of fracture surface from basalt FRP after tensile failure in the mid-span. In Figure 7a, no obvious gap between the fiber and the matrix was observed, which indicated a good interfacial bond between the fiber and the matrix. The close-up image of the fiber/epoxy interface in Figure 7b shows that only a small amount of epoxy remained on the basalt fiber after the tensile failure of BFRP strengthened wood beams. This indicates that the fiber was pulled out from epoxy matrix during the test. The reasons can be the smooth surface of the basalt fiber or the low wetting behavior between epoxy and basalt fiber. Similar pull-out failure can be also found in FFRP and GFRP. Therefore, methods to increase the surface roughness of fiber (e.g., with alkali solution for flax [7]) or to improve the wetting behavior between fiber and polymer are possibilities that could improve the interface bond between fibers and polymer in FRP composites.

## 4. Conclusions

This study presented the structural behavior of wood beams externally strengthened with various FRP composites. The effects of fabric materials, FRP thicknesses and the sequence of arrangement of the FRP laminas on the flexural behavior of FRP strengthened wood beams were investigated through three-point bending tests. It was shown that the load bearing capacity of the beam under bending was increased as the number of FRP layers increased. The beam strengthened with HFRP had an average higher maximum deflection before failure, yet relatively similar maximum bending loading and elastic modulus compared to the ones strengthened with single type FRPs. It was also observed that the failure modes of FRP strengthened wood beams changed from tensile failure to FRP debonding as the number of layers and maximum bending load increased. This was an indication that the interface between epoxy and wood became more decisive as the FRP became stronger. Under the light microscope, air bubbles were observed in the FRP, which may create inhomogeneity and stress concentration in the cross section of the FRP and could have led to the premature failure of the FRP and the whole beam structure. Under scanning electron microscope, fiber pull-out failure was observed at fracture area of the FRP. The failure was suspected mainly due to the combined smooth surface and the low wetting behavior of the fiber. Improvement can be made by increasing the surface roughness and by improving the wetting properties of the fiber.

## Figures and Tables

**Table 1:** Matrix of the specimens.

Specimen	Name ^1^	Number of the FRP Laminates	Number of Replications
Wood	W	0	3
FFRP–wood	W_1L-F	1	3
	W_2L-F	2	3
	W_3L-F	3	3
BFRP–wood	W_1L-B	1	3
	W_2L-B	2	3
	W_3L-B	3	3
GFRP–wood	W_1L-G	1	3
	W_2L-G	2	3
	W_3L-G	3	3
Hybrid–wood	W_3L-GBF	3	3
	W_3L-BFG	3	3
	W_3L-BGF	3	3

1. W for wood; L for layers; B, G and F for basalt, glass and flax, respectively.

**Table 2:** Testing result of flat coupon tensile test and standard three-point bending test of FRP laminates.

Name ^1^	Number of Replications	Nominal Fiber Thickness ^2^	Thickness	Elastic Modulus	Strength	Strain at Peak Load
		mm	mm	GPa	MPa	%
	Tensile span of extensometer = 140 mm, testing speed = 2.5 mm/min					
1L_B_Te	10	0.7	1.11	6.2 (± 0.8)	49.6 (± 8.2)	0.92 (± 0.22)
2L_B_Te	9	1.4	3.11	6.1 (± 0.7)	61.1 (± 9.4)	1.15 (± 0.15)
3L_B_Te	10	2.1	3.41	6.0 (± 0.4)	56.3 (± 6.1)	1.03 (± 0.12)
1L_F_Te	10	1.2	1.81	4.8 (± 0.3)	41.7 (± 5.5)	1.29 (± 0.31)
2L_F_Te	8	2.4	3.07	5.4 (± 0.2)	48.2 (± 1.7)	1.30 (± 0.07)
3L_F_Te	6	3.6	4.33	5.6 (± 0.1)	76.8 (± 2.1)	1.69 (± 0.12)
1L_G_Te	10	0.9	1.06	19.3 (± 1.5)	377.1 (± 55.7)	2.12 (± 0.68)
2L_G_Te	5	1.7	1.71	23.3 (± 0.7)	493.6 (± 46.0)	2.18 (± 0.29)
3L_G_Te	10	2.6	2.72	22.4 (± 1.0)	449.1 (± 38.8)	2.09 (± 0.47)
	Bending span = 100 mm; testing speed = 1%/min, maximum strain before stop = 5%					
1L_B_Be	10	0.7	1.11	5.8 (± 0.5)	79.6 (± 7.2)	2.07 (± 0.24)
2L_B_Be	10	1.4	3.11	6.3 (± 0.5)	156.8 (± 11.5)	2.74 (± 0.15)
3L_B_Be	10	2.1	3.41	5.8 (± 0.5)	139.9 (± 11.8)	2.65 (± 0.18)
1L_F_Be	9	1.2	1.81	3.7 (± 0.7)	60.3 (± 10.0)	2.26 (± 0.36)
2L_F_Be	10	2.4	3.07	5.1 (± 0.2)	94.6 (± 7.1)	3.37 (± 0.25)
3L_F_Be	10	3.6	4.33	4.8 (± 0.2)	90.3 (± 3.0)	3.23 (± 0.23)
1L_G_Be	10	0.9	1.06	8.0 (± 0.6)	90.4 (± 6.8)	1.90 (± 0.20)
2L_G_Be	10	1.7	1.71	18.1 (± 2.6)	331.0 (± 31.3)	2.80 (± 0.15)
3L_G_Be	10	2.6	2.72	16.9 (± 2.1)	525.0 (± 50.9)	4.18 (± 0.36)

1. L for layer; B, F, G for basalt, flax and glass, respectively; Te and Be for tensile and bending, respectively. 2. Approximated nominal fiber thicknesses. The values depend on the pressure applied during the measurement and the different weaving structures of the fabrics.

**Table 3:** Test results and relevant improvements of three-point bending test on wood beams.

FRP Type	Layer	Name	Maximum Load Capacity	Load Capacity Improvement	Elastic Modulus	Elastic Modulus Improvement	Maximum Mid-Span Deflection	Deflection Improvement
			Fmax	D_F_	E	D_E_	D	D_d_
			kN	%	GPa	%	Mm	%
None	0	W	2.8 (± 0.8)	------	9.0	------	12.7 (± 1.0)	------
Flax	1	W_1L-F	4.5 (± 0.6)	60.7	12.7	40.5	16.4 (± 2.6)	29.1
	2	W_2L-F	5.5 (± 0.3)	96.4	12.6	39.8	18.8 (± 6.0)	48.0
	3	W_3L-F^1^	6.2 (± 0.0)	121.4	12.9	42.4	21.2 (± 3.2)	66.9
Basalt	1	W_1L-B^1^	3.2 (± 0.3)	14.3	10.1	12.0	21.8 (± 8.6)	71.7
	2	W_2L-B	4.2 (± 0.7)	50.0	10.0	10.5	21.5 (± 7.3)	69.3
	3	W_3L-B	5.8 (± 0.3)	107.1	11.0	21.7	20.7 (± 3.1)	63.0
Glass	1	W_1L-G	4.8 (± 0.8)	71.4	9.2	1.8	34.2 (± 3.7)	169.3
	2	W_2L-G	6.1(± 0.1)	117.9	13.3	46.9	31.1 (± 6.9)	144.9
	3	W_3L-G	6.5 (± 0.4)	132.1	15.1	66.6	26.8 (± 5.1)	111.0
Hybrid	3	W_3L-BFG	5.6 (± 0.5)	100.0	13.9	53.3	28.1 (± 4.0)	121.3
	3	W_3L-BGF	5.8 (± 0.3)	107.1	14.6	61.8	30.8 (± 5.8)	142.5
	3	W_3L-GBF	5.9 (± 0.4)	110.7	11.4	26.6	29.8 (± 1.9)	134.6

1: one test from each group were not successfully tested.

**Table 4:** General failure modes for control and FRP strengthened wood beams.

FRP Type		Number of FRP Layers			
		0	1	2	3
without FRP		Tensile failure	---	---	---
FFRP		---	Tensile failure	Tensile failure	Debonding at mid-span
BFRP		---	Tensile failure	Tensile failure	Tensile failure
GFRP		---	Debonding at edge	Debonding at edge	Debonding at edge
HFRP	W_3L-BFG	---	---	---	Debonding at edge
	W_3L-BGF	---	---	---	Debonding at edge
	W_3L-GBF	---	---	---	Tensile failure

## Supplementary Materials

The following are available online at https://www.mdpi.com/2073-4360/11/8/1255/s1.
