# Supplementary material for: Flax, Basalt, E-Glass FRP and Their Hybrid FRP Strengthened Wood Beams: An Experimental Study
Source: Polymers (Basel). 2019 Jul 29;11(8):1255. doi: 10.3390/polym11081255 (PMC6723195; doi:10.3390/polym11081255)
Supplement: Supplementary file 1 [file polymers-11-01255-s001.pdf]

Supplementary Materials

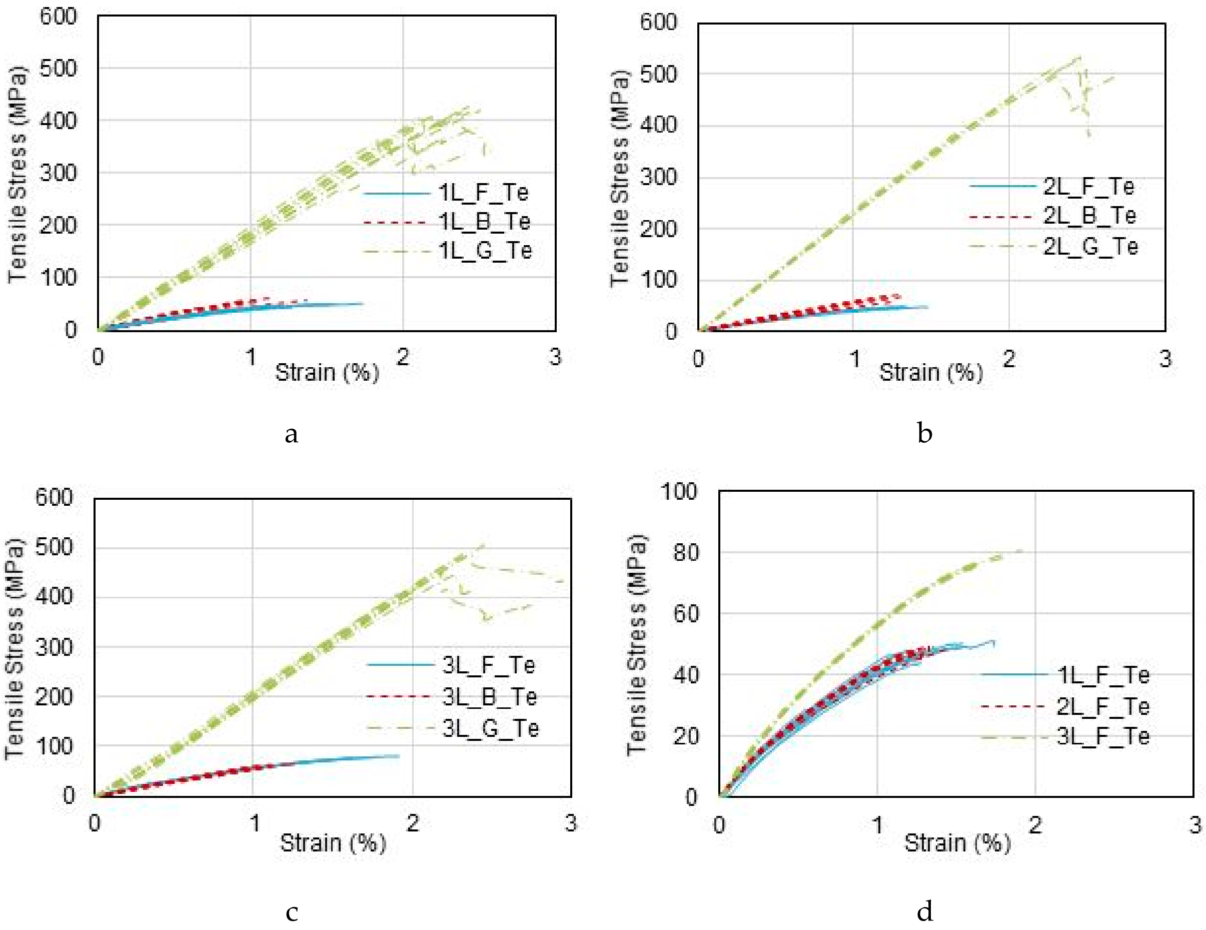

Figure S1 **Stress-strain curve of flat coupon tensile test for (a) 1-layer; (b) 2-layer and (c) 3-layer FRP as well as (d) the layer effect of FFRP**

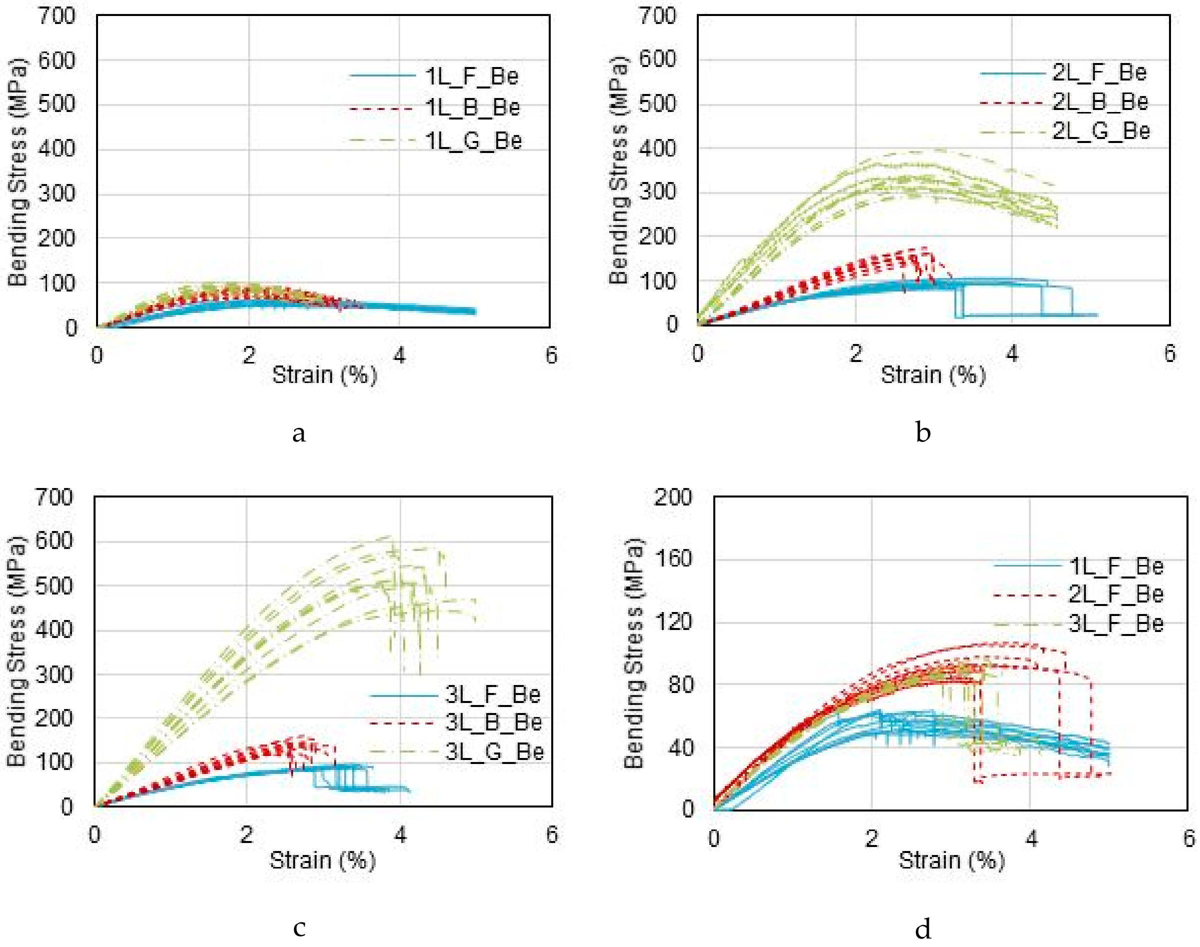

Figure S2 **Stress-strain curve of standard bending test for (a) 1-layer; (b) 2-layer and (c) 3-layer FRP as well as (d) the layer effect of FFRP**

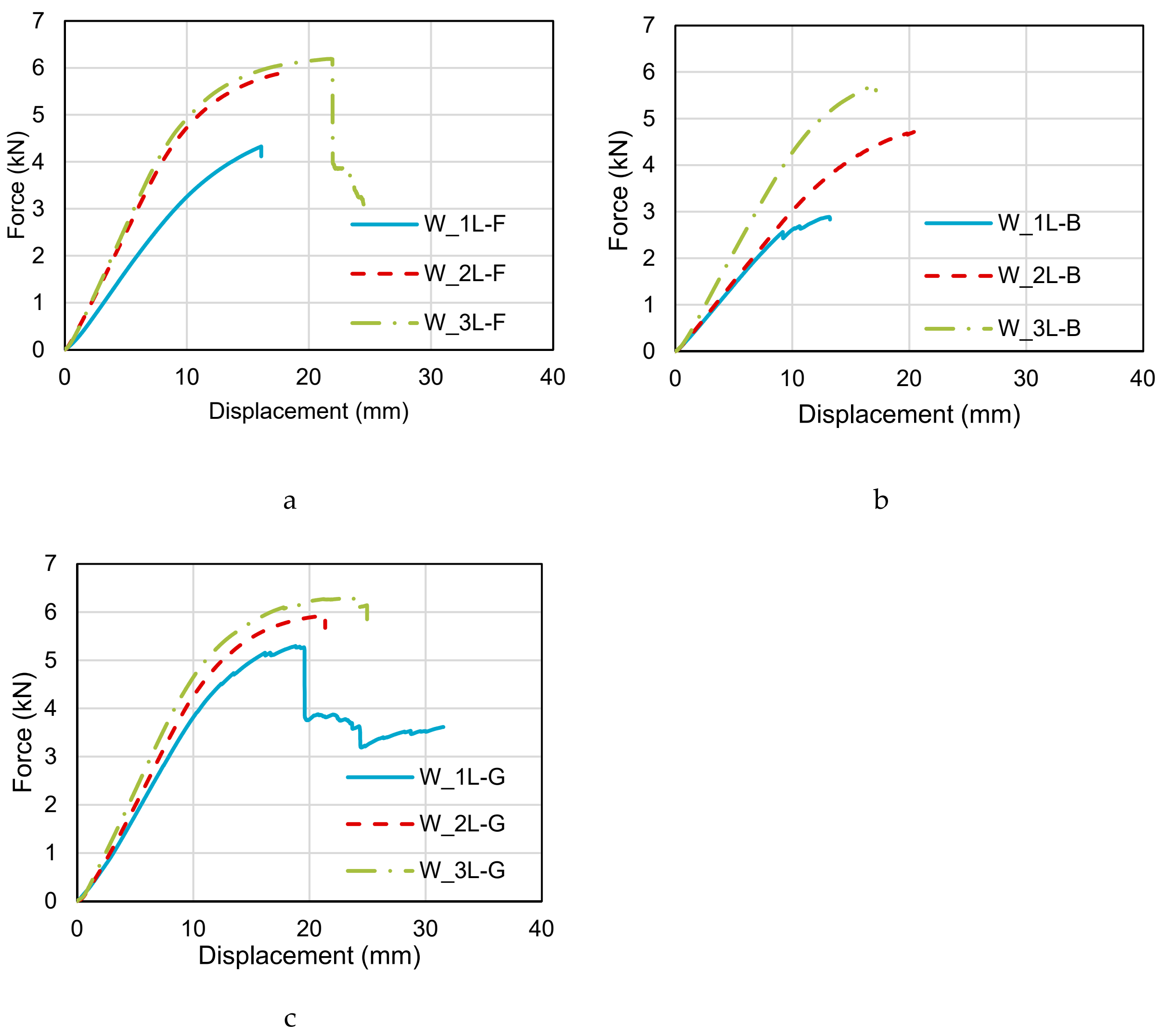

Figure S3 Load-displacement curves of FRP strengthened wood beams with different FRP fabric materials: (a) FFRP, (b) BFRP and (c) GFRP
